# Is ‘Green’ Gold and Silver Nanoparticle Synthesis Environmentally Friendly?

**DOI:** 10.3390/nano15141095

**Published:** 2025-07-14

**Authors:** Lucas Reijnders

**Affiliations:** IBED (Institute for Biodiversity and Ecosystem Dynamics), Faculty of Science, University of Amsterdam, Science Park 904, 1090 GE Amsterdam, The Netherlands; l.reijnders@uva.nl

**Keywords:** ‘green’ synthesis, gold nanoparticles, silver nanoparticles, ecofriendly, environment(ally) friendly

## Abstract

In scientific literature biosynthesis of gold and silver nanoparticles and synthesis of these nanoparticles using small organic molecules such as citrate have been called: ‘green’. It has also been often stated that ‘green’ synthesis of gold and silver nanoparticle is environment(ally) friendly or ecofriendly. The characterization environment(ally) friendly or ecofriendly is commonly comparative. The comparison is between ‘green’ and ‘chemical’ synthesis. The few available comparative life cycle assessments addressing the environmental impacts of ‘green synthesis’ of Ag and Au nanoparticles, if compared with ’chemical’ synthesis, strongly suggest that a ‘green’ synthesis should not be equated with being environment(ally) friendly or ecofriendly. The term ‘green’ for Au and Ag nanoparticles obtained by ‘green’ synthesis is a misnomer. There is a case for only using the terms ecofriendly or environment(ally) friendly for nanoparticle synthesis when there is a firm basis for such characterization in comprehensive comparative cradle-to-nanoparticle life cycle assessment, taking into account the uncertainties of outcomes.

## 1. Introduction

One of the ways to synthesize gold or silver nanoparticles is based on the reduction of a gold or silver salt (commonly HAuCl_4_, AgNO_3_) by strong reductants such as hydrazine (N_2_H_4_) and sodium borohydride (NaBH_4_). The negative impacts of these reductants and of chemical substances used ln the coating of silver and gold nanoparticles has led to much research focussing on the use of more benign reductants and coatings in gold and silver nanoparticle synthesis [1,2]. The results thereof are rather often described in terms of ‘green nanoparticle synthesis’. This is illustrated by Table 1 which includes the results of the title search in the Core Collection of the Web of Science for the ‘green synthesis’ of silver and gold nanoparticles. In the title search about 36% of the scientific papers dealing with the synthesis of silver nanoparticles regarded ‘green synthesis’ and the corresponding percentage regarding gold nanoparticles was about 20%. ‘Green’ synthesis of Ag and Au nanoparticles is an active field. From 30 May 2024 to 30 May 2025, in the Core Collection of the Web of Science, the average monthly increase of titles with ‘green synthesis of silver nanoparticles’ was 36, and of titles with ‘green synthesis of gold nanoparticles’ 5. There are occasionally titles of scientific papers dealing with ‘green’ synthesis of silver or gold nanoparticles in which the product is characterized as ‘green silver nanoparticles’ [3,4,5,6,7,8,9,10,11,12,13,14,15,16] or ‘green gold nanoparticles’ [17,18,19,20,21,22,23,24].

There are two main strands in ‘green’ synthesis of gold and silver nanoparticles. Firstly, there is the use of small organic molecules serving as reductant. This type of synthesis is occasionally called ‘green’ in scientific literature. Secondly, and as to the number of published studies more importantly, there is biological synthesis or biosynthesis of gold and silver nanoparticles. This type of synthesis is often called ‘green’.

Research regarding nanoparticle synthesis, using small organic molecules started long before the term ‘green’ emerged in scientific papers about nanoparticle synthesis. The use of citrate as a reductant for HAuCl_4_ was pioneered by Turkevich et al. [25] and published in 1951. It served the synthesis of (near-)spherical gold colloids coated with citrate in an aqueous solution near boiling point. The Turkevich method can also be used to generate Ag nanoparticles from AgNO_3_ [26]. In scientific literature, citrate used in the synthesis of silver or gold nanoparticles has been characterized as ‘green’ [1,27,28,29], but also as ‘chemical’ [30,31]. This shows that the terms in practice lack precision. The citrate-based synthesis of gold nanoparticles has been developed and modified to generate a variety of shapes and sizes [29,32,33]. Other small organic molecules used as reductants in the synthesis of silver or gold nanoparticles are various amino acids [34,35], vitamin B [1], glucose [36,37], gallic acid [38,39] and ascorbate [40,41]. Amino acids, ascorbic acid, gallic acid and glucose can also serve as coating or capping agents for nanoparticles [38,42,43].

Biosynthesis of Ag and Au nanoparticles may utilize bacteria, fungi, microalgae, viruses, plants and substances derived from bacteria, fungi, algae and plants [2,5,9,44,45,46,47,48,49,50,51,52,53,54,55,56,57,58]. In biosynthesis, reductants present in, or derived from, organisms are used to generate Au and Ag nanoparticles from respectively HAuCl_4_ and AgNO_3_. Additionally, in the biosynthesis of gold and silver nanoparticles substances present in, or derived from, organisms can serve as nanoparticle coatings [53,57,59,60]. Within the field of nanoparticle biosynthesis, studies using plant extracts are by now dominant [61].

In scientific publications ‘green’ Ag and Au nanoparticle synthesis is often characterized as ecofriendly, respectively environment(ally) friendly (see the all fields search in Table 1). The terms ecofriendly and environment(ally) friendly are commonly considered to be synonyms [62]. Their use is not precise: the terms may indicate that the synthesis has no environmental burden or has a minimal environmental burden [62]. The term may also be used in a comparative sense [62]. For instance, a synthesis is environmentally friendly because it has, in comparison with other ways to synthesize the product, a lower environmental burden [62]. In Section 2, a brief description is given of the method used in preparing this manuscript. Section 3 will deal with the questions in what sense the terms environment(ally) friendly and ecofriendly are used, and whether ‘green’ synthesis of Ag and Au nanoparticles is indeed ecofriendly or environment(ally) friendly. Section 4 will present the conclusions of this paper and addresses future directions.

## 2. Method

Databases of major publishers of scientific literature dealing with nanomaterials (ACS, Elsevier, Frontiers, IOP Publishing, MDPI, RSC, Sage, Springer-Nature, Taylor and Francis, Wiley), the Web of Science core collection and Google Scholar have been searched for publications since 1950 relevant to the subjects raised in this paper.

## 3. Is ‘Green’ Synthesis of Gold and Silver Nanoparticles Ecofriendly or Environment(ally) Friendly?

In recent scientific papers claiming that biosynthesis of gold and silver nanoparticles is environment(ally) friendly or ecofriendly [8,9,19,30,53,56,57,63,64,65,66,67,68,69,70,71,72,73,74,75,76,77,78,79,80,81,82,83,84,85,86,87,88,89,90,91,92,93,94] the terms are used in a comparative sense, comparing ‘green ’synthesis with ‘chemical’ synthesis. A recurrent theme is that substances used in ‘green’ synthesis are preferable if compared with substances used in ‘chemical’ synthesis. The latter are characterized as hazardous, having a negative impact on the environment or the ecosystem, dangerous, not environmentally benign, harmful, polluting, respectively toxic or having toxic by-products or wastes.

Replacing substances used in ‘chemical’ synthesis by biological substances, or small organic molecules may be conducive to applications in biomedicine, but is not the only determinant of environmental burden relevant to comparisons with ‘chemical’ synthesis. The following matters may also be important to such comparisons. The first is the input of energy in ‘green’ nanoparticle synthesis. ‘Chemical’ synthesis with the reductants N_2_H_4_ and NaBH_4_ can take place at room temperature [95,96,97]. Synthesis at room temperature may also apply to ‘green’ synthesis, but longer reaction times for ‘green’ synthesis, if compared with ‘chemical’ synthesis, may lead to an increase in the cradle-to-nanoparticle environmental burden linked to energy use for agitation (stirring), lighting and temperature control [1,98]. Also, temperatures may be higher than room temperature. The ‘green’ citrate-based method to generate Au nanoparticles developed by Turkevich et al. [25] used boiling water. Elevated temperatures (up to 121 °C) are furthermore used in ‘green’ synthesis of gold and silver nanoparticles using plant-derived substances [85,91,99,100,101,102,103] and fungal extracts or filtrates [104,105,106]. Also, when (macro)algal extracts are used, ‘green’ synthesis of gold and silver nanoparticles at elevated temperatures has been reported [50,107,108,109]. Reaction times in ‘green’ synthesis may be shortened by ultrasound- and microwave-assisted synthesis [90,110,111]. Such shortening can require substantial energy inputs [112,113]. Papers dealing with the ‘green’ biosynthesis of silver nanoparticles [114,115] have included energy-intensive calcination [116]. Use of energy-intensive liquid phase plasma technology [117] has been reported for the ‘green’ synthesis of silver and gold nanoparticles [118,119], and (high-energetic) gamma radiation for the biosynthesis of gold and silver nanoparticles [120,121].

Secondly, the synthesis of gold and silver nanoparticles results in non-product outputs, containing Au and Ag [122]. If compared with ‘chemical’ synthesis, ‘green’ synthesis of silver and gold nanoparticles may have relatively large non-product outputs of Ag and Au [1,28,123]. Lower yields in ‘green’ synthesis if compared with ‘chemical’ synthesis would, ceteris paribus, lead to a relatively large environmental burden per unit of mass for nanoparticles produced by ‘green’ synthesis, if compared with ‘chemical’ synthesis. This is not necessarily the case, as shown by the high percentage of product yield from HAuCl_4_ for citrate-based Au nanoparticle synthesis [33], see also Table 2. As to biosynthesis of Ag and Au nanoparticles quantified product yields (% of Ag or Au input) are rarely reported. However, recent publications have been found giving percentual yields for optimized laboratory-scale biosynthesis of gold and silver nanoparticles (see Table 2), which are lower than reported percentual yields for optimized ‘chemical’ synthesis.

From the studies presented in Table 2 percentual non-product outputs of Ag and Au in optimized nanoparticle biosynthesis can be derived. For gold nanoparticles the reported non-product Au outputs are in 27.3–37.5% range [111,124] and for silver nanoparticles the reported Ag non-product output is 46.7% [103]. An environmentally sensible way to deal with Au and Ag in non-product outputs is functional recycling: use of the non-product output as starting material for the synthesis of functional gold and silver nanomaterials [122,128]. No example of such recycling following ‘green’ Ag and Au nanoparticle synthesis could be found in scientific literature, though there are studies regarding the functional recycling of non-product outputs containing Ag and Au following ‘chemical’ Ag and Au nanoparticle synthesis [122]. In the absence of recycling, shortfalls of available bulk Ag and Au have to be made up with Ag and Au originating in mining, which are associated with large cradle-to-metal environmental burdens, if compared with recycled Ag and Au [129].

Thirdly: when microorganisms, or substances derived thereof [129,130,131] are used in nanoparticle synthesis, cultivation and cultivation media can have a substantial impact on the cradle-to-nanoparticle environmental burden [129,132,133,134,135]. Cultivating plants to obtain plant-derived substances for the biosynthesis and coating of nanoparticles may also be associated with substantial environmental burdens [136,137,138,139,140,141]. In view of the variety of factors that may impact the environmental burden, cradle-to-nanoparticle comprehensive comparative assessments of environmental burdens using life cycle assessment (LCA) methodology would seem a proper way to determine whether a ‘green’ synthesis of Ag and Au nanoparticles is indeed ecofriendly or environment(ally) friendly, if compared with a ‘chemical’ synthesis (also: [61]).

From searching, as outlined in Section 2, a few scientific studies emerged evaluating the comparative characterization of ecofriendly or environment(ally) friendly for ‘green’ synthesis of Ag or Au nanoparticles, from-cradle-to-nanoparticle. The studies used environmental life cycle assessment methodology (e.g., [142]). In life cycle assessment, comparisons are made on the basis of using the same functional unit. A functional unit is in practice a quantitative aspect of the product and may for instance be mass-based (e.g., 1 g of product consisting of silver nanoparticles) or regarding functionality (e.g., catalytic activity in terms of 1 katal or 1 mole converted per second). The LCA studies to be discussed here used the synthesis of a specified mass of nanoparticles as functional unit for comparing different syntheses.

Pati et al. [1] studied the laboratory-scale synthesis of Au nanoparticles from HAuCl_4_ using a variety of reductants. The impact categories of the LCA considered by Pati et al. [1] are in Table 3 and the reductants studied in Table 4. They found that the ‘green’ reductants do not necessarily reduce the cradle-to-nanoparticle environmental burden of Au nanoparticle synthesis, if compared with the chemical reductants [1]. As to the laboratory-scale synthesis of Au nanoparticles using sodium borohydride and 13 ‘green’ reductants, the cumulative energy demand for the synthesis with sodium borohydride was found by Pati et al. [1] to be lower than the corresponding value for the syntheses using citrate or glucose. The use of organism-derived reductants often led to a higher cumulative energy demand than the use of sodium borohydride [1]. In life cycle assessments of products using a wide range of environmental impact categories cumulative energy demand is a major determinant of the overall environmental burden [143]. Pourzahedi and Eckelman [27] compared the laboratory-scale synthesis of Ag nanoparticles from AgNO_3_ using the reductants borohydride, ethylene glycol, citrate and starch, using cradle-to-nanoparticle environmental assessment covering a range of environmental interventions (see Table 3). They found that the synthesis using borohydride as a reductant had overall a lower environmental burden than the syntheses applying the reductants citrate and soluble starch.

Interestingly, Pourzahedi and Eckelman [27] also found that the production of bulk silver starting with mining, the main source of bulk silver [129], dominated all impact categories of the cradle-to-silver-nanoparticles environmental burden, contributing up to >90% of the burden. Similar conclusions were drawn by Bafana et al. [112] and by Han et al. [129]. In view thereof, it seems farfetched to characterize Ag nanoparticles as ‘green’ [3,4,5,6,7,8,9,10,11,12,13,14,15,16] because the conversion of AgNO_3_ into Ag nanoparticles, which has only a small overall contribution to the life cycle environmental burden of Ag nanoparticles, is characterized as ‘green’. Also, when ’green’ is attributed to products the term is commonly considered to be interchangeable with environment(ally) friendly or eco-friendly [62], which is at variance with the findings of Pourzahedi and Eckelman {27]. In view thereof, it can be concluded that characterizing silver nanoparticles obtained by ’green synthesis as ‘green’ is a misnomer. The characterization ‘green gold nanoparticles’ [17,18,19,20,21,22,23,24] would appear to be even more of a misnomer because the life cycle environmental burden of bulk gold obtained from mining is more than a factor 10 larger than the lifecycle environmental burden of silver obtained from mining [144].

Sierra et al. [123] performed a comparative environmental LCA (with the impact categories presented in Table 3) for the ‘ecofriendly’ laboratory-scale synthesis of silver nanoparticles using leaf extracts from agricultural plants and the corresponding chemical synthesis using sodium borohydride. The assessment was partly cradle-to-washed-nanoparticles. The environmental burden of agricultural production for the supply of leaves was not included. The overall environmental burden of the ‘ecofriendly’ synthesis of Ag nanoparticles, as found by Sierra et al. [123], was larger than the burden of the synthesis using sodium borohydride. Han et al. [129] compared emerging methods for the synthesis of Ag nanoparticles from AgNO_3_ using polyethyleneimine, leaf extract from *Annona glabra* and *Rhodococcus* bacteria, applying a prospective LCA considering the industrial scale and a large range of environmental impacts (see Table 3). They found that the prospective industrial-scale synthesis using polyethyleneimine was linked to a lower cradle-to-nanoparticle greenhouse gas emission than the syntheses using leaf extract from *Annona glabra* and *Rhodococcus*, whereas leaf extract was linked with a relatively high cradle-to-nanoparticle marine eutrophication impact, and *Rhodococcus* with a relatively high cradle-to-nanoparticle use of resources. Han et al. [129] concluded that using leaf extract from *Annona glabra* and *Rhodococcus* bacteria does not guarantee an environmentally friendlier outcome.

Two comments may be made about the comparative life cycle assessments presented here. Firstly, per unit of product mass, the environmental burdens of laboratory-scale syntheses, as studied by Pati et al. [1], Pourzahedi and Eckelman [27] and Sierra et al. [123], may differ substantially from the environmental burdens of industrial-scale production. This is suggested for Ag nanoparticles by the study of Han et al. [129] comparing laboratory-scale and prospective industrial syntheses and applying sensitivity analysis. This is also suggested for gold nanoparticles by comparing the results of Pati et al. [1] for laboratory-scale synthesis of gold nanoparticles using citrate with the prospective LCA study of Grimaldi et al. [145] regarding an industrial-scale synthesis of gold nanoparticles using citrate. It should be noted, though, that anticipatory or prospective life cycle assessments are characterized by relatively large uncertainties [146], a matter not addressed in the study of Grimaldi et al. [145]. Secondly, it may be argued that for comparative studies a functional unit for life cycle assessment that focuses on functionality per unit of mass of the nanoparticles synthesized (e. g. catalytic activity as katal) would be more suitable than a functional unit purely based on mass [61,147]. It should be noted, though, that in nanoparticle biosynthesis the reproducibility of functionality (and size, morphology and coating) is as yet problematical [54,93,148,149,150].

Still, the comparative studies of Pati et al. [1], Pourzahedi and Eckelman [27], Sierra et al. [123] and Han et al. [129] strongly suggest that ’green’ syntheses of Ag and Au nanoparticles should not be equated with environment(ally) friendly or ecofriendly. There is a case for only using the terms ecofriendly or environment(ally) friendly as to Ag and Au nanoparticle synthesis in scientific papers when there is a firm basis for such characterization in comprehensive cradle-to-nanoparticle environmental assessment, taking into account the uncertainties of outcomes.

## 4. Conclusions and Future Directions

The few available comparative life cycle assessments addressing the environmental impacts linked to ’green synthesis’ and ’chemical’ synthesis of Ag and Au nanoparticles strongly suggest that being ’green’ should not be equated with being environment(ally) friendly or ecofriendly. In view thereof, there is a case for using the terms ecofriendly or environment(ally) friendly regarding gold and silver nanoparticle synthesis only when there is a firm basis for this characterization in comprehensive cradle-to-nanoparticle environmental assessment, while taking account of uncertainties. Characterizing gold and silver nanoparticles as ’green’ when they are obtained by ’green’ synthesis is a misnomer. There is a case for researchers to focus on what can actually be done to lower the life cycle environmental burden of Ag and Au nanoparticles, making them more environment(ally) friendly. Implementing options to reduce the life cycle environmental burden may be conducive to the social acceptance of nanoparticle applications (e.g., [151]). Comprehensive life cycle assessment may be helpful in reducing the life cycle environmental burden of nanoparticles [1,27,128,132,147,152,153]. Higher product yields, recycling of non-product outputs, improved resource-efficiency and substitution of fossil fuel-based energy by solar energy are examples of options that may reduce life cycle environmental burdens. To facilitate comprehensive cradle-to-nanoparticle assessment, improvement of the database for nanoparticle biosynthesis is a matter of importance. In view thereof it is preferable that future publications presenting biosynthesis of Ag and Au nanoparticles include (so far often unreported) quantitative data about product yields (as % of noble metal input), product functionality per unit of mass, reproducibility and fate of non-product outputs.

## Figures and Tables

**Table 1:** Results of searches in the Core Collection of the Web of Science (consulted 30 May 2025) referred to in this paper.

Scope of Search	Words Used in Search	Number of Hits
Title	‘green synthesis silver nanoparticles’	2184
Title	‘green synthesis gold nanoparticles’.	648
All fields	‘ecofriendly green synthesis silver nanoparticles’	607
All fields	‘environmentally friendly green synthesis silver nanoparticles’	1411
All fields	‘ecofriendly green synthesis gold nanoparticles’	279
All fields	‘environmentally friendly green synthesis gold nanoparticles’	690

**Table 2:** Percentual product yields for optimized laboratory-scale synthesis of gold and silver nanoparticles from HAuCl_4_ and AgNO_3_.

Type of Synthesis	Nanoparticle	Product Yield (% of Ag or Au Input)	References
Biosynthesis	Au (mostly spherical)	62.5–72.7%	[111,124]
‘Chemical’ synthesis	Au (various shapes)	Up to 95%	[125]
Citrate-based synthesis, with traces tannins	Au (quasi-spherical)	>90%	[33]
Biosynthesis	Ag (roughly spherical)	53.3%	[103]
‘Chemical’ synthesis	Ag (crystalline nanoparticles, nanowires)	>95%, 99%	[126,127]

**Table 3:** Impact categories studied in the comparative life cycle assessments discussed in this paper.

Study	Environmental Impact Categories	Nanoparticles and Scale
Pati et al. [1]	Cumulative Energy Demand Global Warming Potential Metal Depletion Potential Agricultural Land Occupation Fresh Water Ecotoxicity	Gold nanoparticles, laboratory-scale
Pourzahedi and Eckelman [27]	Global Warming Potential Ozone Depletion Potential Oxidizing Smog Formation Respiratory Effects Acidification Human Toxicity Ecotoxicity	Silver nanoparticles, laboratory-scale
Sierra et al. [123]	Abiotic depletion Global Warming Ozone layer Depletion Human Toxicity Fresh Water Toxicity Terrestrial Ecotoxicity Photochemical Oxidation Acidification Eutrophication	Sliver nanoparticles, laboratory-scale
Han et al. [129]	Fossil fuel extraction Ore extraction Water consumption Agricultural land occupation Global warming Ozone layer depletion Ionizing radiation Fresh water eutrophication Fresh water ecotoxicity Human carcinogenicity Non-carcinogenic human toxicity Marine eutrophication Marine ecotoxicity Particulate matter formation Photochemical oxidant formation Terrestrial eutrophication Terrestrial ecotoxicity	Silver nanoparticles, prospective industrial scale and laboratory-scale

**Table 4:** Reductants considered in the lifecycle assessment studies of Pati et al. [1], Pourzahedi and Eckelman [27], Sierra et al. [123] and Han et al. [129].

Study	Chemical Reductants	Small Organic Molecules (‘Green’)	Organism-Derived Reductants and Organisms (‘Green’)
Pati et al. [1]	Sodium borohydride Hydrazine	Citrate Vitamin B D-glucose	C. alba extract C. camphora Cinnamon Coriander Cypress leaf extract Ginseng Grape pomace Mushroom extract Soybean seed extract Sugarbeet pulp
Pourzahedi and Eckelman [27]	Sodium borohydride Ethylene glycol	Citrate	Soluble Starch
Sierra et al. [123]	Sodium borohydride		Plant extract
Han et al. [129]	Polyethyleneimine		Plant extract from Annona glabra Rhodococcus

## Data Availability

Data supporting the findings are available in the references of the paper.

## References

[1] Pati P., McGinnis S., Vikesland P.J. (2014). Life cycle assessment of green nanoparticle synthesis methods. Environ. Eng. Sci..

[2] Singh P., Kim Y., Zhang D., Yang D. (2016). Biological synthesis of nanoparticles from plants and microorganisms. Trends Biotechnol..

[3] Barua S., Konwarh R., Bhattacharya S.S., Das P., Devi K.S.P., Maiti T.K., Mandal M., Karak N. (2013). Non-hazardous anticancerous and antibacterial colloidal ‘green’ silver nanoparticles. Colloids Surf. B Biointerface.

[4] Nakkala J.R., Mata R., Gupta A.K., Sadras S.R. (2014). Biological activities of green silver nanoparticles synthesized with *Acorous calamus* rhizome extract. Eur. J. Med. Chem..

[5] Mohammadlou M., Maghsoudi H., Jafarizadeh-Malmiri M. (2016). A review of green silver nanoparticles based on plants: Synthesis, potential applications and ecofriendly approach. Int. Food Res. J..

[6] Bin-Jumah M.N., Al-Abdan M., Al-Basher G., Alarifi S. (2020). Effects of green silver nanoparticles on apoptosis and oxidative stress in normal and cancerous human hepatic cells in vitro. Int. J. Nanomed..

[7] Hamida R.S., Ali M.A., Goda D.A., Khalil M.I., Redhwan A. (2020). Cytotoxic effect of green silver nanoparticles against ampicillin-resistant Klebsiella pneumoniae. RSC Adv..

[8] Abd-Elsalam K.A. (2022). Green Synthesis of Silver Nanoparticles.

[9] Ronavari A., Igaz N., Adamecz D.I., Szerencses B., Molnar C., Konya Z., Pfeiffer I., Kiricsi M. (2021). Green silver and gold nanoparticles: Biological synthesis approaches and potentials for biomedical applications. Molecules.

[10] Bapat M.S., Singh H., Shukla S.K., Singh P.P., Vo D.-V.N., Yadav A., Goyal A., Sharma A., Kumar D. (2022). Evaluating green silver nanoparticles as prospective biopesticides: An environmental standpoint. Chemosphere.

[11] Aman S., Kaur N., Mittal D., Sharma D., Shukla K., Singh B., Sharma A., Siwal S.S., Thakur V.K., Joshi H. (2023). Novel biocompatible green silver nanoparticles efficiently eliminates multidrug resistant nosocomial pathogens and Mycobacterium species. Indian J. Microbiol..

[12] Sawalha H., Ahmad S.A., Shaharuddin N.A., Sanusi R., Azzeme M., Naganthran A., De Silva C., Abiri R. (2024). Antibacterial activity of green silver nanoparticles on the in vitro pathogen infected *Eucalyptus pellita* plant. Plant Cell Tissue Organ Cult..

[13] Abizi-Moqadam A., Mortazavi-Derazkola S., Zare-Bidaki M., Osmani F., Alizadeh L. (2025). In vitro investigation of antimicrobial and antioxidant properties of green silver nanoparticles synthesized using Ephedra gerardiana plant extract. Curr. Pharm. Biotechnol..

[14] Kaliappan K., Nagarajan P., Jayabalan J., Pushparaj H., Elumalai S., Paramanathan B., Manickam V., Jang H.T., Mani G. (2025). Systematic antimicrobial, biofilm, free radical inhibition and Tyrosinase inhibition assessments of efficient green silver nanoparticles from the aqueous root extract of *Cyphostemma adenocaule* (CA). RSC Pharm..

[15] Koohestani H., Nabilu M., Balooch A. (2025). Biosynthesis and investigation of antibacterial properties of green silver nanoparticles using fruit extracts of Wild barberry, Medlar (*Mespilus germanica* L.) and Hawthorn. Food Chem. Adv..

[16] Pattnaik C., Mishra R., Lenka A., Kar B.P., Dash S.K., Sahoo L.N., Tripathy S.K., Nayak G.C., Sahoo S. (2025). Exploring the versatility of carbohydrate capped green silver nanoparticles as multi-dimensional reagents for targeted applications. J. Mol. Struct..

[17] Wu F., Zhu J., Li G., Wang J., Veeraraghavan V.P., Mohan S.K., Zhang Q. (2019). Biologically synthesized green gold nanoparticles from *Siberian ginseng* induce growth-inhibitory effect on melanoma cells (B16). Artif. Cells Nanomed. Biotechnol..

[18] Bin-Jumah M.N., Al-Abdan M., Al-Basher G., Alarifi S. (2020). Molecular mechanism of cytotoxicity, genotoxicity and anticancer potential of green gold nanoparticles on human liver normal and cancerous cells. Dose-Response.

[19] Mikhailova E. (2021). Gold nanoparticles: Biosynthesis and potential of biomedical application. J. Funct. Biomater..

[20] De Lima Oliveira E.G., Viera S.A., Da Silva F.A.G., Da Costa M.M., Gomes A.S.L., De Oliveira H.P. (2022). Synergistic antibacterial activity of green gold nanoparticles and tannin-based derivatives. BioChem.

[21] Mitri N., Rahme K., Fracasso G., Ghanem E. (2022). Upgrading gold to green nanoparticles: Applications in prostate cancer. Adv. Nat. Sci. Nanosci. Nanotechnol..

[22] Sargazi S., Laraib U., Er S., Rahdar A., Hassanisaadi M., Zafar M.N., Diez-Pascual A.M., Bilal M. (2022). Application of green gold nanoparticles in cancer therapy and diagnosis. Nanomaterials.

[23] Ariski R., Lee K.K., Kim Y., Lee C. (2024). The impact of pH and temperature on the green gold nanoparticles preparation using Jeju Hallabong peel extract for biomedical applications. RSC Adv..

[24] Kelleci K. (2025). Potential of green gold nanoparticles synthesized from *Punica granatum* extract on Leishmaniasis and breast cancer treatment. ChemistrySelect.

[25] Turkevich J., Stevenson P.C., Hillier J. (1951). A study of the nucleation and growth processes in the synthesis of colloidal gold. Discuss. Faraday Soc..

[26] Gorup L.F., Longo E., Leite E.R., Camargo E.R. (2011). Moderating effect of ammonia on particle growth and stability of quasi monodisperse silver nanoparticles synthesized by the Turkevich method. J. Colloid Interface Sci..

[27] Pourzahedi L., Eckelman M.J. (2015). Comparative life cycle assessment of silver nanoparticle synthesis routes. Environ. Sci. Nano.

[28] Bhattarai B., Zaker Y., Bigioni T.P. (2018). Green synthesis of gold and silver nanoparticles: Challenges and opportunities. Curr. Opin. Green Sustain. Chem..

[29] Memon A.G., Channa I.A., Shaikh A.A., Ahmad J., Soomro A.F., Giwa A.S., Baig Z.T., Mahdi W.A., Alshehri S. (2022). Citrate-capped AuNP fabrication, characterization and comparison with commercially produced nanoparticles. Crystals.

[30] Firdhouse M.J., Lalitha P. (2022). Biogenic green synthesis of gold nanoparticles and their applications: A review of promising properties. Inorg. Chem. Commun..

[31] Tessema B., Gonfa G., Hailegiorgis S.M., Prabhu S.V., Manivannan S. (2023). Synthesis and characterization of silver nanoparticles using reducing agents of bitter leaf (*Vernonia amygdalina*) extract and tri-sodium citrate. Nano-Struct. Nano-Objects.

[32] Kimling J., Maier M., Okenve B., Kotaidis V., Ballot H., Plech A. (2006). Turkevich method for gold nanoparticle synthesis revisited. J. Phys. Chem. B.

[33] Piella J., Bastus N.G., Puntes V. (2016). Size-controlled synthesis of sub-10-nanometer citrate-stabilized gold nanoparticles and related optical properties. Chem. Mater..

[34] Maruyama T., Fujimoto Y., Maekawa T. (2015). Synthesis of gold nanoparticles using various amino acids. J. Colloid Interface Sci..

[35] De Matos R.A., Courrol L.C. (2017). Biocompatible silver nanoparticles prepared with amino acids and a green method. Amino Acids.

[36] Ghorbani H.R. (2015). Green synthesis of gold nanoparticles. Orient. J. Chem..

[37] Putri G.E., Gusti F.R., Sary A.N., Zainul R. (2019). Synthesis of silver nanoparticles used chemical reduction method by glucose as reducing agent. J. Phys. Conf. Ser..

[38] Li D., Liu Z., Yuan Y., Liu Y., Niu F. (2015). Green synthesis of gallic acid-coated silver nanoparticles with high anti-microbial activity and low cytotoxicity to normal cells. Process Biochem..

[39] Nunez R.N., Veglia A.V., Pacioni N.L. (2018). Improving reproducibility between batches of silver nanoparticles using an experimental design approach. Microchem. J..

[40] Cumberland S.A., Lead J.R. (2013). Synthesis of NOM-capped silver nanoparticles: Size, morphology, stability, and NOM binding characteristics. ACS Sustain. Chem. Eng..

[41] Pinheiro L.D.S.M., Sangoi G.G., Vizzotto B.S., Ruiz Y.P.M., Galembeck A., Pavoski G., Espinosa D.C.R., Machado A.K., da Silva W.L. (2024). Silver nanoparticles from ascorbic acid: Biosynthesis, characterization, in vitro safety profile, antimicrobial activity and phytotoxicity. Mater. Chem. Phys..

[42] Carnovale C., Bryant G., Shukla R., Bansal V. (2019). Identifying trends in gold nanoparticle toxicity and uptake: Size, shape, capping ligand, and biological corona. ACS Omega.

[43] Vukoje I., Lazic V., Sredojevic D., Fernandes M.M., Lanceros-Mendez S., Ahrenkiel S.P., Nedeljkovic J.M. (2022). Influence of glucose, sucrose and dextran coatings on the stability and toxicity of silver nanoparticles. Int. J. Biol. Macromol..

[44] Annamalai J., Nallamuthu T. (2015). Characterization of biosynthesized gold nanoparticles from aqueous extract of *Chlorella vulgaris* and their anti-pathogenic properties. Appl. Nanosci..

[45] Banerjee K., Rai V.R. (2018). A review on mycosynthesis, mechanism, and characterization of silver and gold nanoparticles. BioNanoScience.

[46] El-Seedi H.R., El-Shabasy R.M., Khalifa S.A.M., Saeed A., Shah A., Shah R., Iftikhar F.J., Abdel-Daim M.M., Omri A., Hajrahand N.H. (2019). Metal nanoparticles fabricated by green chemistry using natural extracts: Biosynthesis, mechanisms and applications. RSC Adv..

[47] Roy A., Bulut O., Some S., Mandal A.K., Yilmaz M.D. (2019). Green synthesis of silver nanoparticles: Biomolecule-nanoparticle organisations targeting anti-microbial activity. RSC Adv..

[48] Tepale N., Fernandez-Escamilla V.V.A., Carreon-Alvarez C., Gonzalez-Coronel V.J., Luna-Flores A., Carreon-Alvarez A., Aguilar J. (2019). Nanoengineering of gold nanoparticles: Green synthesis, characterization, and applications. Crystals.

[49] Bano S., Agrawal M., Pal R. (2020). A review of green synthesis of silver nanoparticles through plant extract and its medicinal applications. J. Indian Chem. Soc..

[50] Costa L.H., Hemmer J.V., Wanderlind E.H., Gerlach O.M.S., Santos A.L.H., Tamanaha M.S., Bella-Cruz A., Correa R., Bazani H.A.G., Radetski C.M. (2020). Green synthesis of gold nanoparticles obtained from algae *Sargassum cymosum*: Optimization, characterization and stability. BioNanoScience.

[51] Slepicka P., Kasalkova N.S., Siegel J., Kolska Z., Svorcik V. (2020). Methods of gold and silver nanoparticles preparation. Materials.

[52] Kumar J.A., Krithiga T., Manigandan S., Sathish S., Renita A.A., Prakash P., Prasad B.S.N., Kumar T.R.P., Rajasimman M., Hosseini-Bandegharaei A. (2021). A focus on green synthesis of metal/metal based oxide nanoparticles: Various mechanisms and applications towards ecological approach. J. Clean. Prod..

[53] Restrepo C.V., Villa C.C. (2021). Synthesis of silver nanoparticles, influence of capping agents, and dependence on size and shape: A review. Environ. Nanotechnol. Monitor. Manag..

[54] Balestri A., Cardellini J., Berti D. (2023). Gold and silver nanoparticles as tools to combat multidrug-resistant pathogens. Curr. Opin. Colloid Interface Sci..

[55] Salem S.S., Soliman M.K.Y., Azab M.S., Abu-Elghait M. (2024). Optimization growth conditions of *Fusarium pseudonygamai* for mycosynthesized gold and silver nanoparticles using response surface methodology. BioNanoScience.

[56] Ahmed B., Tahir M.B., Sagir M., Hassan M. (2024). Bio-inspired sustainable synthesis of silver nanoparticles as next generation of nanoproduct in antimicrobial and catalytic applications. Mater. Sci. Eng. B.

[57] Vadakkan K., Rumjit N.P., Ngangbam A.K., Vijayanand S., Nedumpillil N.K. (2024). Novel advancements in the sustainable green synthesis approach of silver nanoparticles (AgNPs) for antibacterial therapeutic applications. Coord. Chem. Rev..

[58] Ohja S., Khan A., Sahoo C.R., Mohapatra R.K., Tripathi D.K., Mukherjee M., Guldhe A., Nayak M. (2025). A review on biosynthesis of nanoparticles via microalgal technology and their biomedical applications. BioNanoScience.

[59] Chugh G., Singh B.R., Adholeya A., Barrow C.J. (2022). Role of proteins in the biosynthesis and functioning of metallic nanoparticles. Crit. Rev. Biotechnol..

[60] Xu F., Li Y., Zhao X., Liu G., Pang B., Liao N., Li H., Shi J. (2024). Diversity of fungus mediated synthesis of gold nanoparticles: Properties, mechanisms, challenges, and solving methods. Crit. Rev. Biotechnol..

[61] Garcia-Quintero A., Palencia M. (2021). A critical analysis of environmental sustainability metrics applied to green synthesis of nanomaterials and the assessment of environmental risks associated with the nanotechnology. Sci. Total Environ..

[62] Reijnders L. (2024). Are magnesium alloys applied in cars sustainable and environmentally friendly? A Critical Review. Sustainability.

[63] Behzad F., Naghib S.M., Kouhbanani M.A.J., Tabatabaei S.N., Zare Y., Rhee K.Y. (2021). An overview of the plant mediated green synthesis of noble metal nanoparticles for antibacterial applications. J. Ind. Eng. Chem..

[64] Hatipoglu A. (2021). Rapid green synthesis of gold nanoparticles: Synthesis, characterization and antimicrobial activity. Progr. Nutr..

[65] Kim B., Song W.C., Park S.Y., Park G. (2021). Green synthesis of silver and gold nanoparticles via *Sargassum serratifolium* extract for catalytic reduction of organic dyes. Catalysts.

[66] Padalia H., Chanda S. (2021). Antioxidant and anticancer activities of gold nanoparticles synthesized using aqueous leaf extract of *Ziziphus nummularia*. BioNanoScience.

[67] Shreyash N., Bajpai S., Khan M.A., Vijay Y., Tiwary S.K., Sonker M. (2021). Green synthesis of nanoparticles and their biomedical applications: A review. ACS Appl. Nano Mater..

[68] Ankudze B., Samlafo V.B. (2022). Repeated use of *Cyperus esculentus* tubers, towards sustainable green synthesis of silver nanoparticles. BioNanoScience.

[69] Bergal A., Matar G.H., Andac M. (2022). Olive and green tea leaf extracts mediated green synthesis of silver nanoparticles (AgNPs): Comparison investigation on characterization and antibacterial activity. BioNanoScience.

[70] Majoumouo M.S., Tincho M.B., Yimta Y.D., Adekiya T.A., Aruleba R.T., Ayawei N., Boyom F.F., Morris T. (2022). Biosynthesis of silver nanoparticles using *Bersama engleriana* fruits extracts and their potential inhibitory effect on resistant bacteria. Crystals.

[71] Mousavi-Khattat M., Nourbakhshan H., Afrazeh S., Aminorroaya S.H., Shakeran Z. (2022). Donkey dung-mediated synthesis of silver nanoparticles and evaluation of their antibacterial, antifungal, anticancer, and DNA cleavage activities. BioNanoScience.

[72] Santhosh P.B., Genova J., Chamati H. (2022). Green synthesis of gold nanoparticles: An eco-friendly approach. Chemistry.

[73] Abuzeid H.M., Julien C.M., Zhu L., Hashem A.M. (2023). Green synthesis of nanoparticles and their energy storage, environmental, and biomedical applications. Crystals.

[74] Dharmadhas J.S., Danaraj J., Packiavathy I.A.S.V. (2023). Green fabrication of *Nerium oleander*-mediated silver nanomaterials: Synthesis structural, and stability analysis. BioNanoScience.

[75] Naser P.T.N., Thoppil J.E. (2023). Synthesis, characterisation and antimicrobial efficacy of gold and silver nanoparticles from fruit extracts of *Ficus drupacea* Thunb. var. pubescens. BioNanoScience.

[76] Patil T., Gambhir R., Vibhute A., Tiwari A.P. (2023). Gold nanoparticles, synthesis methods, functionalization and biological applications. J. Cluster Sci..

[77] Rabbi F., Ahmad I., Nisar A., Rauf A., Alshammari A., Alharbi M., Suleria H.A.R. (2023). Synthesis, characterization and antibacterial assessment (synergism) of silver nanoparticles prepared with stem bark extract of *Sterculia diversifolia*. Crystals.

[78] Rahman H., Rauf A., Khan S.A., Ahmad Z., Alshammari A., Alharbi M., Alam A., Suleria H.A.R. (2023). Green synthesis of silver nanoparticles using *Rhazya stricta* Decne extracts and their anti-microbial and anti-oxidant activities. Crystals.

[79] Ritu, Verma K.K., Das A., Chandra P. (2023). Phytochemical-based synthesis of silver nanoparticle: Mechanism and potential applications. BioNanoScience.

[80] Rizki I.N., Klaypradit W., Patmawati (2023). Utilization of marine organisms for the green synthesis of silver and gold nanoparticles and their applications: A review. Sustain. Chem. Pharm..

[81] Arshad F., Naikoo G.A., Hassan I.U., Chava S.R., El-Tanani M., Aljabali A.A., Tambuwala M.M. (2024). Bioinspired and green synthesis of silver nanoparticles for medical applications: A green perspective. Appl. Biochem. Biotechnol..

[82] Asefian S., Ghavam M. (2024). Green and environmentally friendly synthesis of silver nanoparticles with antibacterial properties from some medicinal plants. BMC Biotechnol..

[83] De Jesus R.A., de Assis G.C., de Oliveira R.J., Costa J.A.S., da Silva C.M.P., Iqbal H.M.N., Ferreira L.F.R. (2024). Metal/metal oxide nanoparticles: A revolution in the biosynthesis and medical applications. Nano-Sruct. Nano-Objects.

[84] Fakharzadeh M., Fazljoo S.E. (2024). Biosynthesis of silver nanoparticles using *Barley straw* and *Fumaria parviflora* extract: Optimization of synthesis, evaluating of nanoparticles stability, antibacterial activities. BioNanoScience.

[85] Jamil Y.M.S., Al-Hakimi A.N., Al-Maydama H.M.A., Almahwiti G.Y., Qasem A., Saleh S.M. (2024). Optimum green synthesis, characterization and antibacterial activity of silver nanoparticles prepared from an extract of *Aloe fleurentinorum*. Int. J. Chem. Eng..

[86] Khedr W.E., Shaheen M.N.F., Elmahdy E.M., El-Bendary M.A., Hamed A.A., Mohamedin A.H. (2024). Silver and gold nanoparticles: Ecofriendly synthesis, antibiofilm, antiviral, and anticancer bioactivities. Prep. Biochem. Biotechnol..

[87] Nejad M.S., Tarighi S., Taheri P., Darroudi M. (2024). Extracellular biosynthesis silver nanoparticles Using *Streptomyces* spp. and evaluating its effects against important tomato pathogens and cancer cells. BioNanoScience.

[88] Paulo E.A., de Souza C.M., de Souza N.A.A., Quirino J.N., Furlaneto-Maia L., Furlaneto M.C. (2024). One-pot and environmentally friendly biosynthesis of silver nanoparticles from *Enterococcus durans*: Activity against fluconazole-resistant pathogenic *Candida tropicalis*. BioNanoScience.

[89] Sakore P., Bhattacharya S., Belemkar S., Prajapati B.G., Elossaily G.M. (2024). The theranostic potential of green nanotechnology-enabled gold nanoparticles in cancer: A paradigm shift on diagnosis and treatment approaches. Results Chem..

[90] Edo G.I., Mafe A.N., Ali A.B.M., Akpoghelie P.O., Yousif E., Isoje E.F., Igbuku U.A., Ismael S.A., Essaghah A.E.A., Ahmed D.S. (2025). Green biosynthesis of nanoparticles using plant extracts, mechanisms, advances, challenges, and applications. BioNanoScience.

[91] Ghoreishi S.M., Mortazavi-Derazkola S. (2025). Ecofriendly synthesis of gold nanoparticles via *tangerine* peel extract: Unveiling their multifaceted biological and catalytic potential. Heliyon.

[92] Omole R.K., Agboluaje E.O., Torimiro N., Xiong M.P., Adeyemi O.I., George R.C., Daramola O.B., Muthupandian S. (2025). Intracellular production of gold nanoparticles from *Lysinibacillus fusiformis,* their spectro-structural characterization and antibacterial potential on MDR bacteria of chronic wounds. BioNanoScience.

[93] Shahzadi S., Fatima S., ul ain Q., Shafiq Z., Janjua M.R.S.A. (2025). A review on green synthesis of silver nanoparticles (SNPs) using plant extracts: A multifaceted approach in photocatalysis, environmental remediation, and biomedicine. RSC Adv..

[94] Wehbe N., Mesmar J.E., El Kurdi R., Al-Sawalmih A., Badran A., Patra D., Baydoun E. (2025). *Halodule uninervis* extract facilitates the green synthesis of gold nanoparticles with anticancer activity. Sci. Rep..

[95] Sivaraman S.K., Kumar S., Santhanam V. (2010). Room temperature synthesis of gold nanoparticles—Size control by slow addition. Gold Bull..

[96] Dong P.V., Ha C.H., Binh L.T., Kasbohm J. (2012). Chemical synthesis and antibacterial activity of novel-shaped silver nanoparticles. Int. Nano Lett..

[97] Streszewski B., Jaworski W., Paclawski K., Csapó E., Dékány I., Fitzner K. (2012). Gold nanoparticles formation in the aqueous system of gold (III)chloride complex ions and hydrazinesulfate–kinetic studies. Colloids Surf. A Physicochem. Eng. Asp..

[98] Leng W., Pati P., Vikesland P.J. (2015). Room temperature seed-mediated growth of gold nanoparticles: Mechanistic investigations and life cycle assessment. Environ. Sci. Nano.

[99] Mohammadlou M., Jafarizadeh-Malmiri H., Maghsoudi H. (2017). Hydrothermal green synthesis of silver nanoparticles using *Pelargonium/Geranium* leaf extract and evaluation of their antifungal activity. Green Process. Synth..

[100] Satpathi S., Patra A., Ahirwar B., Hussain M.D. (2020). Process optimization for green synthesis of gold nanoparticles mediated by extract of *Hygrophila spinosa* T. Anders and their biological applications. Phys. E Low-Dimens. Syst. Nanostruct..

[101] Singh A., Gaud B., Jaybhaye S. (2020). Optimization of synthesis parameters of silver nanoparticles and its anti-microbial activity. Mater Sci. Energy Technol..

[102] Srivastava P., Gunawan C., Soeriyadi A., Amal R., Hoehn K., Marquis C. (2021). In vitro coronal protein signatures and biological impact of silver nanoparticles synthesized with different natural polymers as capping agents. Nanoscale Adv..

[103] Ghasemi S., Dabirian S., Kariminejad F., Koohi D.E., Nemattalab M., Majidimoghadam S., Zamani E., Yousefbeyk F. (2024). Process optimization for green synthesis of silver nanoparticles using *Rubus discolor* leaves extract and its biological activities against multi-drug resistant bacteria and cancer cells. Sci. Rep..

[104] Saxena J., Sharma P.K., Sharma M.M., Singh A. (2016). Process optimization for green synthesis of silver nanoparticles by *Sclerotinia sclerotiorum* MTCC 8785 and evaluation of its antibacterial properties. SpringerPlus.

[105] Eskandari-Nojedehi M., Jafarizadeh-Malmiri H., Rahbar-Shahrouzi J. (2018). Hydrothermal green synthesis of gold nanoparticles using mushroom (*Agaricus bisporus)* extract: Physico-chemical characteristics and antifungal activity studies. Green Process. Synth..

[106] Alves M.F., Murray P.G. (2022). Biological synthesis of Monodisperse uniform-size silver nanoparticles (AgNPs) by fungal cell-free extracts at elevated temperature and pH. J. Fungi.

[107] Aboelfetoh E.F., El-Shenody R.A., Ghobara M.M. (2017). Ecofriendly synthesis of silver nanoparticles using green algae (*Caulerpa serrulata)* reaction optimization, catalytic and antibacterial activities. Environ. Monit. Assess..

[108] Mohamed R.M., Fawzy E.M., Shehab R.A., Abdel-Salam M.O., Salah El Din R.A., Abd El Fatah H.M. (2022). Production, characterization and cytotoxicity effects of silver nanoparticles from brown alga (*Cystoseira myrica*). J. Nanotechnol..

[109] Hamida R.S., AlMotwaa S.M., Al-Otaibi W.A., Alqhtani H.A., Ali M.A., Bin-Meferij M.M. (2024). Apoptotic induction by biosynthesized gold nanoparticles using *Phormidesmis communis* strain AB_11_10 against osteosarcoma cancer. Biomedicines.

[110] Dash S.S., Banerjee J., Samanta S., Giri B., Dash S.K. (2022). Microwave-assisted fabrication of silver nanoparticles utilizing seed extract of *Areca catechu* with antioxidant potency and evaluation of antibacterial efficacy against multidrug resistant pathogenic bacterial strains. BioNanoScience.

[111] Gao L., Mei S., Ma H., Chen X. (2022). Ultrasound-assisted green synthesis of gold nanoparticles using citrus peel extract and their enhanced anti-inflammatory activity. Ultrason. Sonochem..

[112] Bafana A., Kumar S.V., Temizel-Sekeryan S., Dahoumane S.A., Haselbach L., Jeffryes C.S. (2018). Evaluating microwave-synthesized silver nanoparticles from silver nitrate with life cycle assessment techniques. Sci. Total Environ..

[113] Fuentes-Garcia J.A., Santoyo-Salzar J., Rangel-Cortes E., Goya G.F., Cardozo-Mata V., Pescador-Rojas J.A. (2021). Effect of ultrasonic irradiation power on sonochemical synthesis of gold nanoparticles. Utrason. Sonochem..

[114] Khan J., Naseem I., Bibi S., Ahmad S., Altaf F., Hafeez M., Almoneef M.M., Ahmad K. (2023). Green synthesis of silver nanoparticles (Ag-NPs) using *Debregeasia Salicifolia* for biological applications. Materials.

[115] Priyadarshini S.S., Sethi S., Rout S., Mishra P.M., Pradhan N. (2023). Green synthesis of microalgal biomass-silver nanoparticle composite showing antimicrobial activity and heterogenous catalysis of nitrophenol reduction. Biomass Convers. Biorefin..

[116] Del Pilar Rodriguez-Rojas M., Bustos-Terrones V., Dias-Cardenas M.Y., Vasquez-Vélez E., Martinez H. (2024). Life cycle assessment of green synthesis of TiO_2_ nanoparticles vs chemical synthesis. Sustainability.

[117] Hadinoto K., Rao N.R.H., Astorga J.B., Zhou R., Biazik J., Zhang T., Masood H., Cullen P.J., Prescott S., Henderson R.K. (2023). Hybrid plasma discharges for energy efficient production of plasma-activated water. Chem. Eng. J..

[118] Skiba M., Vorobyova V. (2022). Green synthesis and characterization of silver nanoparticles using *Prunus persica* L. (peach pomace) with natural deep eutectic solvent and plasma liquid process. Chem. Pap..

[119] Skiba M., Vorobyova V. (2025). Green alginate-mediated synthesis of gold nanoparticles by liquid-phase plasma for biomedical and environmental applications: Antimicrobial activity and catalytic hydrogenation of organic dyes. Nanotechnol. Environ. Eng..

[120] El-Batal A.I., Hashem A.-A.M., Abdelbaky N.M. (2013). Gamma radiation mediated green synthesis of gold nanoparticles using fermented soybean-garlic aqueous extract and their antimicrobial activity. SpringerPlus.

[121] Kumar R.M., Rao B.L., Asha S., Narayana B., Byrappa K., Wang Y., Yao D., Sangappa Y. (2015). Gamma radiation assisted biosynthesis of silver nanoparticles and their characterization. Adv. Mater. Lett..

[122] Reijnders L. (2024). Recycling of non-product outputs containing rare elements originating in nanomaterial synthesis. Environ. Sci. Nano.

[123] Sierra M.J., Herrera A.P., Ojeda K.A. (2018). Life cycle analysis of the synthesis of eco-friendly metallic nanoparticles. Contemp. Eng. Sci..

[124] El-Naggar N.E.-A., El-Sawah A.A., Elmansy M.F., Elmessiry O.T., El Saidy M.E., El-Sherbeny M.K., Sarhan M.T., Elhefnawy A.A., Dalal S.R. (2024). Process optimization for gold nanoparticles biosynthesis by *Streptomyces albogriseolus* using artificial neural network, characterization and antitumor activities. Sci. Rep..

[125] Jiang Y., Salley D., Sharma A., Keenan G., Mullin M., Cronin L. (2022). An artificial intelligence enabled chemical synthesis robot for exploration and optimization of nanomaterials. Sci. Adv..

[126] Zhu J., Kan C., Wan J., Han M., Wang G. (2011). High yield synthesis of uniform Ag nanowires with high aspect ratios by introducing the long chain PVP in an improved polyol process. J. Nanomater..

[127] Desireddy A., Conn B.E., Guo J., Yoon B., Barnett R.N., Monahan B.M., Kirschbaum K., Griffith W.P., Whetten R.L., Landman U. (2013). Ultrastable silver nanoparticles. Nature.

[128] Kang S., Rahman A., McGinnis S., Vikesland P. (2024). Toward environmentally favorable nano-sensing by production of reusable gold nanoparticles from gold nanowaste: Life cycle and nanocircular economy implications. Environ. Sci. Nano.

[129] Han Z., Teah H.Y., Hirasawa I., Kikuchi Y. (2025). Prospective life cycle assessment of emerging silver nanoparticle synthesis methods: One pot polyethyleneimine chemical reduction and biological reductions. J. Clean. Prod..

[130] Patil M.P., Kang M., Niyonizigiye I., Singh A., Kim J., Seo Y.B., Kim G. (2019). Extracellular synthesis of gold nanoparticles using the marine bacterium *Paracoccus haeundaensis* BC74171^T^ and evaluations of their antioxidant activity and antiproliferative effect on normal and cancer cell lines. Colloid Surf. B Bionterfaces.

[131] Do J.-M., Hong J.W., Yoon H.-S. (2025). Microalgae-mediated green synthesis of silver nanoparticles: A sustainable approach using extracellular polymeric substances from *Graesiella emersonii* KNUA204. Front. Micobiol..

[132] Wu F., Zhou Z., Hicks A.L. (2019). Life cycle impact of titanium dioxide nanoparticle synthesis through physical, chemical, and biological routes. Environ. Sci. Technol..

[133] Rahman A., Kang S., McGinnis S., Vikesland P.J. (2022). Life cycle impact assessment of iron oxide (Fe_3_O_4_/γ-Fe_2_O_3_) nanoparticle synthesis routes. ACS Sustain. Chem. Eng..

[134] Braud L., McDonnell K., Murphy F. (2023). Environmental life cycle assessment of algae systems: Critical review of modelling approaches. Renew. Sustain. Energy Rev..

[135] Gurreri L., Rindina M.C., Luciano A., Falqui L., Fino D., Mancini G. (2024). Microalgae production in an industrial scale photobioreactor plant: A comprehensive life cycle assessment. Sustain. Chem. Pharm..

[136] Tuomisto H.L., Hodge I.D., Riordan P., MacDonald D.W. (2012). Does organic farming reduce environmental impacts?—A meta-analysis of European research. J. Environ. Manag..

[137] Bessou C., Basset-Mens C., Tran T., Benoist A. (2013). LCA applied to perennial cropping systems: A review focussed on the farm stage. Int. J. Life Cycle Assess..

[138] Hakeem K.R. (2015). Crop Production and Global Environmental Issues.

[139] Reynolds T.W., Waddington S.R., Anderson C.L., Chew A., True Z., Cullen A. (2015). Environmental impacts and constraints associated with the production of major food crops in sub-Saharan Africa and South Asia. Food Secur..

[140] Li Y., Wu W., Yang J., Cheng K., Smith P., Sun J., Xu A.X., Yue Q., Pan G. (2022). Exploring the environmental impact of crop production in China using a comprehensive footprint approach. Sci. Total Environ..

[141] Martin M., Bengtsson E., Carotti L., Orrestig K., Orsini F. (2023). Environmental assessment of greenhouse herb production: A case of longitudinal improvement options in Sweden. Resour. Conserv. Recycl..

[142] Huijbregts M.A.J., Steinman Z.J.N., Elshout P.M.F., Stam G., Verones F., Viera M.D.M., Hollander A., Zijp M., van Zelm R. (2016). ReCiPe 2016.

[143] Huijbregts M.A.J., Hellweg S., Frischknecht R., Hendriks H.W.M., Hungerbühler K., Hendriks A.J. (2010). Cumulative energy demand as predictor for the environmental burden of commodity production. Environ. Sci. Technol..

[144] Nuss P., Eckelman M.J. (2014). Life cycle assessment of metals: A scientific synthesis. PLoS ONE.

[145] Grimaldi F., Pucciarelli M., Gavriilidis A., Dobson P., Lettieri P. (2020). Anticipatory life cycle assessment of gold nanoparticles production: Comparison of milli-continuous flow and batch synthesis. J. Clean. Prod..

[146] Thonemann N., Schulte A., Maga D. (2020). How to conduct prospective life cycle assessment for emerging technologies: A systematic review and methodological guidance. Sustainability.

[147] Nizam N.U.M., Hanafiah M.M., Woon K.S. (2021). A content review of life cycle assessment of nanomaterials: Current practices, challenges and future prospects. Nanomaterials.

[148] Abegunde S.M., Afolayan B.O., Ilesanmi T.M. (2024). Ensuring sustainable plant-assisted nanoparticles synthesis through process standardization and reproducibility: Challenges and future directions—A review. Sustain. Chem. One World.

[149] Almatroudi A. (2024). Unlocking the potential of silver nanoparticles: From synthesis to versatile bio applications. Pharmaceutics.

[150] Kirubakaran D., Wahid J.B.A., Karmegam N., Jeevika R., Sellapillai L., Rajkumar M., Senthilkumar K.J. (2025). A comprehensive review on the green synthesis of nanoparticles: Advancements in biomedical and environmental applications. Biomed. Mater. Devices.

[151] Huang X., Auffan M., Eckelman M.J., Elimelech M., Kim J., Rose J., Zuo K., Li Q., Alvarez P.J.J. (2024). Trends, risks and opportunities in environmental nanotechnology. Nat. Rev. Earth Environ..

[152] Fuentes O.P., Trujillo D.M., Sánchez M.E., Abrego-Perez A.L., Osma J.F., Cruz J.C. (2024). Embracing sustainability in the industry: A study of environmental, economic and exergetic performances in large-scale production of magnetite nanoparticles. ACS Sustain. Chem. Eng..

[153] Han Z., Teah H.Y., Kikuchi Y. (2024). Global sensitivity analysis on system design parameters of silver nanoparticle production. Comput. Aid. Chem. Eng..

